# Antimicrobial activity of fermented Maillard reaction products, novel milk-derived material, made by whey protein and *Lactobacillus rhamnosus* and *Lactobacillus gasseri* on *Clostridium perfringens*

**DOI:** 10.5713/ab.20.0290

**Published:** 2021-02-15

**Authors:** Yujin Kim, Sejeong Kim, Soomin Lee, Jimyeong Ha, Jeeyeon Lee, Yukyung Choi, Hyemin Oh, Yewon Lee, Nam-su Oh, Yohan Yoon, Heeyoung Lee

**Affiliations:** 1 Department of Food and Nutrition, Sookmyung Women’s University, Seoul 04310, Korea; 2 Risk Analysis Research Center, Sookmyung Women’s University, Seoul 04310, Korea; 3 Department of Food and Nutrition, Dong-eui University, Busan 47340, Korea; 4 Department of Food and Biotechnology, Korea University, Sejong 30019, Korea; 5 Food Standard Research Center, Korea Food Research Institute, Wanju 55365, Korea

**Keywords:** Antimicrobial Effects, *Clostridium perfringens*, Fermented Maillard Reaction Products

## Abstract

**Objective:**

The objective of this study was to evaluate the antimicrobial effects of fermented Maillard reaction products made by milk proteins (FMRPs) on *Clostridium perfringens* (*C. perfringens*), and to elucidate antimicrobial modes of FMRPs on the bacteria, using physiological and morphological analyses.

**Methods:**

Antimicrobial effects of FMRPs (whey protein plus galactose fermented by *Lactobacillus rhamnosus* [*L. rhamnosus*] 4B15 [Gal-4B15] or *Lactobacillus gasseri* 4M13 [Gal-4M13], and whey protein plus glucose fermented by *L. rhamnosus* 4B15 [Glc-4B15] or *L. gasseri* 4M13 [Glc-4M13]) on *C. perfringens* were tested by examining growth responses of the pathogen. Iron chelation activity analysis, propidium iodide uptake assay, and morphological analysis with field emission scanning electron microscope (FE-SEM) were conducted to elucidate the modes of antimicrobial activities of FMRPs.

**Results:**

When *C. perfringens* were exposed to the FMRPs, *C. perfringens* cell counts were decreased (p<0.05) by the all tested FMRPs; iron chelation activities by FMRPs, except for Glc-4M13. Propidium iodide uptake assay indicate that bacterial cellular damage increased in all FMRPs-treated *C. perfringens*, and it was observed by FE-SEM.

**Conclusion:**

These results indicate that the FMRPs can destroy *C. perfringens* by iron chelation and cell membrane damage. Thus, it could be used in dairy products, and controlling intestinal *C. perfringens*.

## INTRODUCTION

*Clostridium perfringens*, gram-positive and spore-forming anaerobe, is the third common cause of foodborne illness in United States [1]. Also, this bacterium is responsible for the symptoms, such as abdominal cramps and severe diarrhea, caused by ingestion of food which is contaminated in large number of *C. perfringens*, especially proteinaceous foods, meat, and poultry [2]. Besides, *C. perfringens* generally inhabits in a human gut, and its prevalence in the gut increases, as humans get old [3]. A significant proportion of this bacterium in gut microbiota may be associated with autism in children and neurological disorders, such as neuromyelitis optica [4–6]. The virulence of *C. perfringens* attributes to its toxins, especially *C. perfringens* enterotoxin (CPE) produced during its sporulation in intestines [7]. Due to its ability to bind to claudin receptors, which are junction proteins in intestines, it is known that CPE creates pore on the cell membrane and results in cell death, causing diarrhea and abdominal pain [7]. Thus, this bacterium needs to be controlled. However, there are no edible materials which are applicable to human for destroying *C. perfringens* specifically inhabits in intestines.

In previous studies, glycated milk proteins, such as whey protein glycated with galactose and glucose, were studied for anti-inflammatory activities and their protective effects against oxidative stress [8,9]. Furthermore, fermented Maillard reaction products (FMRPs), which were produced from glycated milk proteins, have recently been introduced for their physiological functions, such as antioxidant, antihypertensive, antithrombotic, and hepatoprotective properties [10,11]. It was reported that antimicrobial activities of Maillard reaction products vary according to the kinds of amino acids [12]. Moreover, Maillard reaction products, which were derived from the foods, such as coffee, beer, and wine, showed different antimicrobial activities [13,14]. Meanwhile, the antimicrobial activity of FMRPs has never been investigated yet to date. Moreover, the effect of Maillard reaction products against the bacteria inhabiting gastrointestinal tract was not elucidated. In order to reduce incidence of diseases due to the pathogenicity of intestinal microbes, and risks such as antibiotics and stress tolerance, it is necessary to study FMRPs possessing physiological and antimicrobial activities. Therefore, the objective of this study was to evaluate the antimicrobial activity of FMRPs against *C. perfringens* and to elucidate the mode of FMRPs for antimicrobial activity.

## MATERIALS AND METHODS

### Preparation of fermented Maillard reaction products

FMRPs were prepared with whey protein (Davisco Foods International Inc., Le Sueur, MN, USA), sugars (galactose or glucose), and lactic acid bacteria (*Lactobacillus rhamnosus* 4B15 or *Lactobacillus gasseri* 4M13) (Table 1). For Maillard reaction, 50 mg/g of whey protein isolate and 25 mg/g of sugar (galactose or glucose) were mixed in deionized water, followed by shaking at 60 rpm in a pilot-scale instrument controlled by a pilot-scale pasteurization unit (Powerpoint International, Tokyo, Japan) at 65°C for 24 h. These Maillard reacted galactose and glucose were then fermented by *L. rhamnosus* 4B15 or *L. gasseri* 4M13 at 6 Log colony-forming unit (CFU)/mL at 37°C for 48 h to prepare FMRPs (Gal-4B15, Gal-4M13, Glu-4B15, and Glu-4M13). The cultures were then centrifuged at 2,000×*g* for 30 min (Hitachi CR-21G rotor R20A, Hitachi Ltd., Tokyo, Japan). Subsequently, the supernatants were transferred into aluminum containers, followed by freeze-drying using a lyophilizer (OPERON FDUT-8608, Gimpo, Gyeonggi, Korea) for 72 h. The freeze-dried FMRPs were powdered and stored at 4°C until further use.

### Bacterial preparation

To evaluate antimicrobial effects of FMRPs on *C. perfringens*, 1 mL of *C. perfringens* strains NCCP15911, NCCP15912, NCCP10846, NCCP10858, NCCP10976, and NCCP10347 were inoculated in 10 mL cooked meat medium (Oxoid, Basingstoke, Hampshire, UK), and the cultures were placed in airtight containers with Anaerogen (Oxoid, UK), followed by incubation at 37°C for 24 h. Then, 0.1 mL aliquots of the cultures were inoculated into 9 mL fresh brain-heart infusion (BHI; BD Difco, Sparks, MD, USA), followed by anaerobic incubation at 37°C for 24 h. The subcultures were mixed, and then centrifuged at 1,912×*g* and 4°C for 15 min. The cell pellets were washed twice with phosphate buffered saline (PBS, pH 7.4; 0.2 g of KH_2_PO_4_, 1.5 g of Na_2_HPO_4_, 8.0 g of NaCl, and 0.2 g of KCl in 1 L of distilled water). The cell suspensions of *C. perfringens* were then diluted to OD_600_ = 0.01 with PBS to obtain 5 to 6 Log CFU/mL of inoculum.

### Antimicrobial effect of fermented Maillard reaction products

To evaluate the antimicrobial effects of FMRPs on *C. perfringens*, a hundred microliters of *C. perfringens* inoculum and 900 μL of FMRPs (100 mg/mL; Gal-4B15, Gal-4M13, Glc-4B15, and Glc-4M13) were mixed in a well of a 24-well plate (SPL Life Sciences, Gyeonggi, Korea), and the plate was placed in an airtight container, containing Anaerogen (Oxoid, UK), followed by anaerobic incubation at 37°C for 24 h. The samples were then serially diluted with buffered peptone water (BPW; BD Difco, USA), and 100 μL of the diluents were spread-plated on tryptose sulfite cycloserine agar (TSC; Oxoid, UK) to enumerate *C. perfringens* cells.

### Iron chelation activity

To evaluate the iron chelation activity by FMRPs, 100 μL of FMRPs (20 mg/mL), 600 μL of distilled water, and 100 μL of 0.2 mM iron (II) chloride tetrahydrate (FeCl_2_·4H_2_O, SAMCHUN chemical Co. Ltd., Seoul, Korea) were mixed, and the mixture reacted for 30 sec at room temperature. Subsequently, 200 μL of 0.1 mM Ferrozine (3-(2-Pyridyl)-5,6-diphenyl-1,2,4-triazine-p,p’-disulfonic acid monosodium salt hydrate; Sigma Aldrich, St. Louis, MO, USA) were suspended in the mixture, and the samples reacted for 10 min at room temperature. Thereafter, turbidity of the samples was measured at 562 nm with a UV-spectrophotometer (OPTIZEN 2120UV, Mecasys Co., Ltd., Daejeon, Korea), and iron chelation activity was calculated as follows;

$$\text{Iron chelation activity }(\%)=\left(1-\frac{\text{sample solution absorbance}}{\text{blank solution absorbance}}\right)\times 100$$

Absorbance of blank solution was measured with distilled water instead of with samples [15].

### Propidium iodide uptake assay

To observe the effects of FMRPs on bacterial cell permeability in short time, propidium iodide (PI) uptake assay for FMRPs-exposed *C. perfringens* was conducted according to the method described by Kim et al [16]. *C. perfringens* were cultured in cooked meat medium at 37°C for 24 h. The bacterial cultures were centrifuged at 10,000×*g* for 1 min at room temperature, and supernatants were discarded. One-milliliter portion of each FMRP sample (200 mg/mL) was transferred to the cell pellets and mixed, followed by incubation at 37°C for 60 min. Subsequently, 50 μL of *C. perfringens* incubated with FMRP were diluted with 950 μL of PBS in a FACS tube (BD Bioscience, San Jose, CA, USA), and 10 μL of PI solution (1 mg/mL) (Sigma Aldrich, USA) was then added to the diluted cultures. The mixtures were analyzed with FACSCanto II (BD Bioscience, USA), and PI activities for *C. perfringens* exposed to FMRPs were calculated, using CellQuest Pro software (BD Bioscience, USA). The percentages of FMRPs-treated bacteria figured in each histogram are relatively calculated to PBS-treated bacteria at a same designated point.

### Morphological observation

To elucidate the antimicrobial activity of FMRPs against *C. perfringens*, morphological observations were conducted, using field emission scanning electron microscope (FE-SEM; JEOL JSM-7600F, JEOL USA Inc., Peabody, MA, USA), according to the method described by Kim et al [16]. *C. perfringens* were cultured in 500 μL BHI containing the glass slides anaerobically at 37°C for 24 h with Anaerogen (Oxoid, UK). After incubation, the glass slides were removed from the cultures, and aseptically transferred to sterile 24-well flat-bottomed polystyrene plates (SPL, Korea). Thirty milliliters of 1.8% glutaraldehyde (Sigma-Aldrich Co., St. Louis, MO, USA) were carefully placed over the glass slides, and left for 30 min at room temperature for the reaction, followed by washing glass slides three times with 1 mL distilled water for 5 min. Subsequently, the glass slides were treated with 2% osmium tetroxide solution (Sigma Aldrich, USA) for 20 min at room temperature for a second fixation, followed by washing them three times with 1 mL distilled water for 5 min. The fixed bacterial cells on the glass slides were gradually dehydrated with 1 mL of 25%, 50%, 75%, 90%, and 100% ethyl alcohol for 5 min. Afterwards, the glass slides were thoroughly dried with 15 μL hexamethyldisilazane (Sigma-Aldrich, USA). The samples were eventually coated with a sputter coater (Cressington 108auto SEM sputter coater, Cressington Scientific Instruments Ltd., Watford, UK), and the bacterial cells were observed, using field emission scanning electron microscopy.

### Statistical analysis

The bacterial cell counts (Log CFU/mL) and iron chelation activity were analyzed by SAS (version 9.2; SAS Institute Inc., Cary, NC, USA), using general linear model procedure. Significant differences of LS-means were analyzed with a pairwise t-test at α = 0.05.

## RESULTS AND DISCUSSION

### Antimicrobial activities of fermented Maillard reaction products

**Figure 1:**
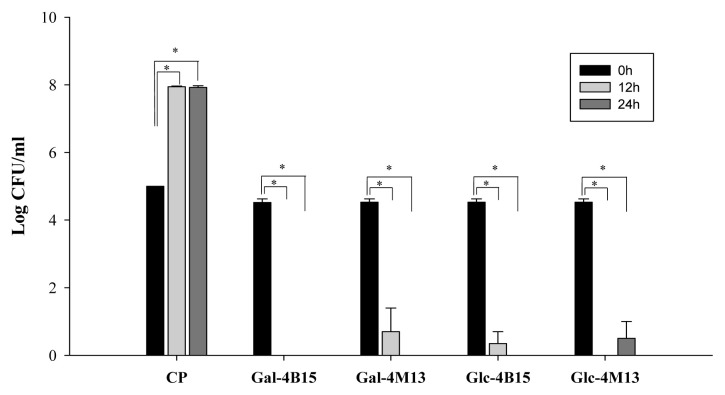
*Clostridium perfringens* cell counts in fermented Maillard reaction products (Gal-4B15, Gal-4M13, Glc-4B15, and Glc-4M13) according to time for 24 h at 37°C. CP, *Clostridium perfringens*; Gal-4B15, Galactose + *L. rhamnosus* 4B15; Gal-4M13, Galactose + *L. gasseri* 4M14; Glc-4B15, Glucose + *L. rhamnosus* 4B15; Glc-4M13, Glucose + *L. gasseri* 4M13. * p<0.05.

*C. perfringens* cell counts in the FMRPs significantly decreased (p<0.05) at 37°C for 12 and 24 h (Figure 1), regardless of FMRPs used. Especially Gal-4B15 reduced *C. perfringens* cell counts below detection limit (10 CFU/mL) even after 12 h of incubation (Figure 1). This result indicates that FMRPs had significant antimicrobial effects against *C. perfringens*. The functional effects of FMRPs, such as antihypertension and prevention of cardiovascular disease, were found in other studies [10,11]. Taken together with the antimicrobial functions of FMRPs against *C. perfringens*, FMRPs may reduce intestinal *C. perfringens* and may be potential materials as health-promoting supplements. Although some fermented dairy products which show antimicrobial activity against *C. perfringens*, such as Kefir and probiotic yogurt, already exist, FMRPs are distinctive due to its antihypertensive and cardiovascular disease-preventive effects [17].

### Iron chelation activity

Theoretically, high iron chelation activity indicates that iron as an essential nutrient for microorganisms in the environment is unavailable to bacteria, which in turn suppresses bacterial growth and decreases bacterial cell counts [18,19]. Gal-4M13 (23.87%) significantly showed high iron chelation activity compared to Glc-4M13 (0.50%) (p<0.05) (Table 2). This suggests that Gal-4M13 may have better iron chelation activities and thus, they may play an important role in inhibiting the growth by limiting uptake of iron in bacterial growth environments. However, only iron chelation activity is not affecting the antimicrobial effects of Gal-4M13 in this study, because Glc-4B15 and Glc-4M13 showed similar antimicrobial effects on *C. perfringens*, although they showed difference in iron chelation activity.

### Propidium iodide uptake assay

**Figure 2:**
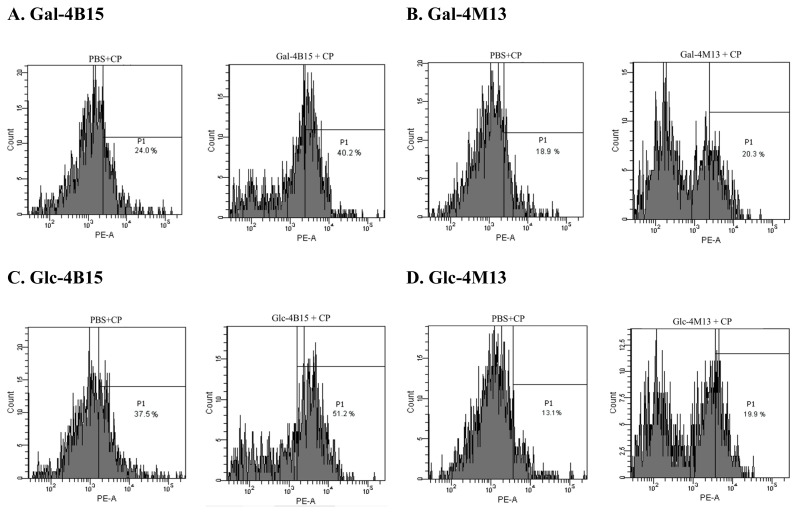
Fluorescent-activated cell sorting histograms of *Clostridium perfringens* exposed to fermented Maillard reaction products. Prior to measure propidium iodide in each treatment, the histograms were adjusted with PBS-treated *C. perfringens* (PBS-CP). PI, propidium iodide; PBS, phosphate-buffered saline; CP, *C. perfringens*; Gal-4B15, Galactose + *L. rhamnosus* 4B15; Gal-4M13, Galactose + *L. gasseri* 4M14; Glc-4B15, Glucose + *L. rhamnosus* 4B15; Glc-4M13, Glucose + *L. gasseri* 4M13.

In PI uptake assay, movement of peaks in a histogram, which represents the number of events detected at particular intensity, to the right indicates that the cell membrane is damaged. In this study, the movement of the peaks to right, and the change in the shape of the peaks and the number of the events were observed after the FMRPs treatment. Movement of fractions towards right X-axis indicates damages of bacterial cells via detection of wavelength of PI which binds to DNA leaked from damaged bacterial cells [20]. The events of treated *C. perfringens* increased from 24.0% to 40.2% by Gal-4B15 and from 18.9% to 20.3% by Gal-4M13. Glc-4B15 and Glc-4M13 treatments also increased the portions of *C. perfringens* from 37.5% to 51.2% and from 13.1% to 19.9%, respectively (Figure 2). This result indicates that FMRPs caused cell membrane damage, and it might be one of the modes to destroy *C. perfringens* cells. To date, physicochemical properties of dairy antimicrobial peptides, such as net charge, amphipathicity, hydrophobicity, etc., have shown effects on interaction with cell membranes and integration of the membranes with the peptides [21]. Therefore, it is assumed that peptides in FMRPs specifically interacted with the bacterial cell membranes and occurred the cell leakage.

### Morphological analysis

**Figure 3:**
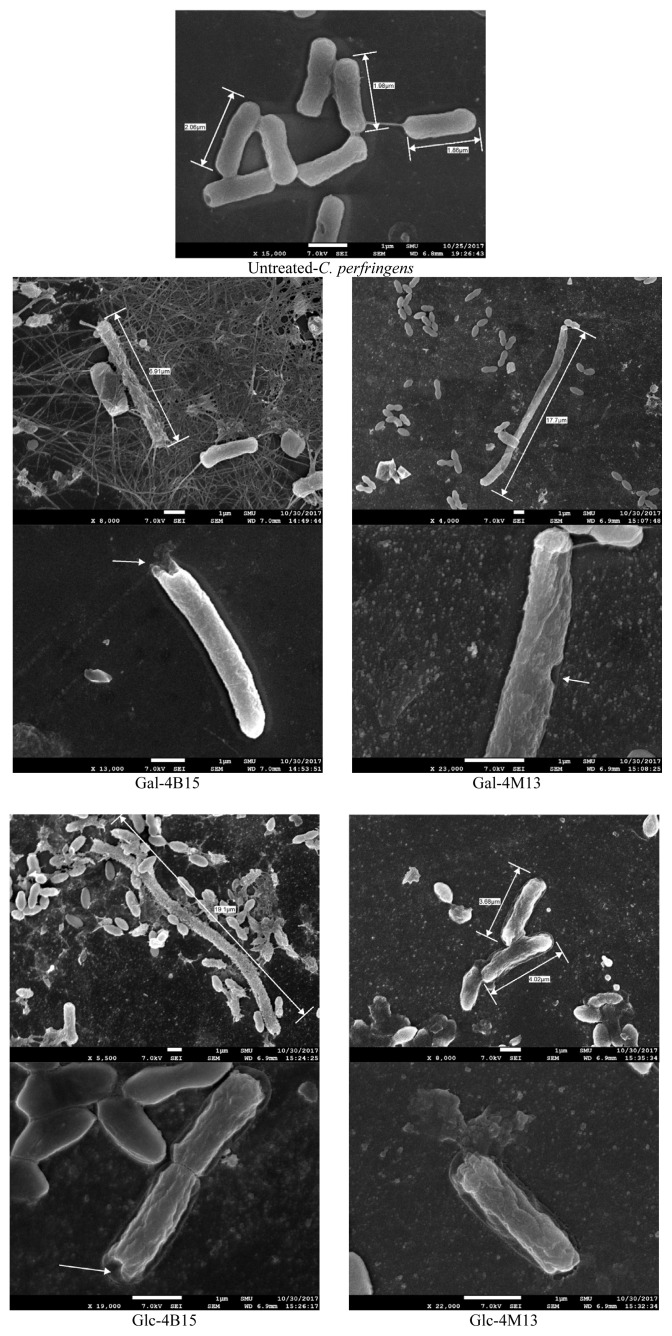
Morphological observation of *Clostridium perfringens*, which was exposed to fermented Maillard reaction products for 24 h, using FE-SEM. Gal-4B15, Galactose + *L. rhamnosus* 4B15; Gal-4M13, Galactose + *L. gasseri* 4M14; Glc-4B15, Glucose + *L. rhamnosus* 4B15; Glc-4M13, Glucose + *L. gasseri* 4M13.

The PI uptake assay showed that FMRP treatments caused cell membrane damages, which were confirmed visually by FE-SEM. FMRP-treated *C. perfringens* had an uneven cell membrane, and pores on the cell membrane surface (Figure 3). In addition, the lengths of the FMRP-treated bacteria increased from 2.0 μm to 12.0 μm in average and 19.1 μm in maximum for *C. perfringens*. Normally each bacteria species consistently maintains their classical bacterial shapes with their complex mechanisms genetically and biochemically [22]. Bacterial cells were found to elongate in order to adjust stressful environments [23]. Everis and Betts [23] also suggested that the length of *Clostridium* spp. increases when they are exposed to stress. When the bacterial cell is exposed to high solute and nutrition concentration, the osmotic forces change the mechanical integrity of bacterial cell and determine the shape [24]. Thus, the elongation of the bacterial cells in this study would be a result of the stressful environment by FMRPs. Taken together with the result of iron chelation activity and PI uptake assay, FMRPs would have antimicrobial activity as they occurred cell membrane damages and iron chelating properties simultaneously.

## CONCLUSION

The FMRPs (Gal-4B15, Gal-4M13, Glc-4B15, and Glc-4M13), which are already known for the functional properties, showed significant antimicrobial activity against *C. perfringens*. The antimicrobial activity of FMRPs would be caused by iron chelation and bacterial cell membrane damage. Moreover, FMRPs contributed to cause stress to *C. perfringens* as its elongation shows. Thus, the FMRPs can be used in various food to control the pathogen. In addition, it can be expected that consumption of FMRPs may control intestinal *C. perfringens* as health-promoting supplements.

## Figures and Tables

**Table 1:** Fermented Maillard reaction products examined for antimicrobial activity on *Clostridium perfringens*, produced by whey protein and sugars fermented by lactic acid bacteria

Milk proteins	Sugars	Lactic acid bacteria	Treatment code
Whey protein	Galactose	*L. rhamnosus* 4B15	Gal-4B15
		*L. gasseri* 4M13	Gal-4M13
	Glucose	*L. rhamnosus* 4B15	Glc-4B15
		*L. gasseri* 4M13	Glc-4M13

**Table 2:** Iron chelation activity (%, mean±standard deviation) of fermented Maillard reaction products

Treatment	Iron chelation activity (%, mean±standard deviation)
Gal-4B15	5.36±3.16^AB^
Gal-4M13	23.87±1.16^A^
Glc-4B15	14.76±12.82^AB^
Glc-4M13	0.50±7.34^B^

A–B Different letters indicate significant differences between means at p<0.05.
